# Assessment of patient safety culture: a nationwide survey of community pharmacists in Kuwait

**DOI:** 10.1186/s12913-018-3662-0

**Published:** 2018-11-22

**Authors:** Fatemah Mohammad Alsaleh, Eman Ali Abahussain, Hamed Hamdi Altabaa, Mohammed Faisal Al-Bazzaz, Noor Barak Almandil

**Affiliations:** 1 Department of Pharmacy Practice, Faculty of Pharmacy, Kuwait University, P.O. Box 24923, 13110 Safat, Kuwait; 2 Department of Clinical Pharmacy Research, Institute for Research and Medical Consultations (IRMC), Imam Abdulrahman Bin Faisal University, P.O. Box 1982, 31441 Dammam, Saudi Arabia

**Keywords:** Safety culture, Community pharmacists, Kuwait

## Abstract

**Background:**

Medication errors have been the largest component of medical errors threatening patient safety worldwide. Several international health bodies advocate measuring safety culture within healthcare organizations as an effective strategy for sustainable safety improvement. To the best of our knowledge, this is the first study conducted in a Middle Eastern country at the level of community pharmacy, to examine safety culture and to evaluate the extent to which patient safety is a strategic priority.

**Methods:**

A descriptive cross-sectional study was conducted. The Pharmacy Survey on Patient Safety Culture (PSOPSC), developed by the Agency for Healthcare Research and Quality (AHRQ), was used to collect data. PSOPSC is a self-administered questionnaire which was previously tested for validity and reliability. The questionnaire was distributed among pharmacists who work in community pharmacies from the five governorates of Kuwait (Capital, Hawalli, Farwaniya, Jahra, and Ahmadi). The Statistical Package for Social Science (SPSS) software, version 24 was used for analysing data.

**Results:**

A total of 255 community pharmacists from the five governorates were approached to participate in the study, of whom 253 returned a completed questionnaire, with the response rate of 99%. Results from the study showed that patient safety is a strategic priority in many aspects of patient safety standards at the level of community pharmacies. This was reflected by the high positive response rate (PRR) measures demonstrated in the domains of “Teamwork” (96.8%), “Organizational Learning-Continuous Improvement” (93.2%) and “Patient Counselling” (90.9%). On the other hand, the lowest PRR was given to the “Staffing, Work Pressure, and Pace” domain which scored 49.7%.

**Conclusions:**

Understanding community pharmacists’ perspectives of patient safety culture within their organization is critical. It can help identify areas of strength and those that require improvement, which can help support decision about actions to improve patient safety. The current study showed that urgent attention should be given to the areas of weakness, mainly in the dimension of “Staffing, Work Pressure and Pace.” The pharmacists pointed the need for adequate breaks between shifts and less distractible work environment to perform their jobs accurately.

**Electronic supplementary material:**

The online version of this article (10.1186/s12913-018-3662-0) contains supplementary material, which is available to authorized users.

## Background

Patient safety, defined as *“the freedom from accidental injuries during the course of medical care; activities to avoid, prevent, or correct adverse outcomes which may result from the delivery of healthcare* [1]*”* is a critical element of healthcare quality and has been a public concern across all healthcare systems worldwide. Medication errors, defined as *“any preventable events that may cause or lead to inappropriate medication use or patient harm while the medication is in the control of the healthcare professional, patient, or consumer”* [2], are a common component of medical errors threatening patient safety [2, 3]. They are estimated to be the 8^th^ leading cause of death in the US and are responsible for the death of approximately 44.000 to 98.000 people every year [1, 4]. While all medication errors are preventable, 28% of Adverse Drug Events (ADE), defined as *“injuries resulting from medical interventions related to a drug”* [2]*,* are preventable and pharmacists can play a pivotal role in preventing 50% of them [5]. Studies have shown that pharmacist intervention can help to improve patient safety and quality of care [6, 7].

Promoting a “culture of safety” within healthcare systems has become one of the major pillars for optimizing patient safety. Following the report from the US Institute of Medicine (IOM) in 1999 [1], there has been a growing recognition within the healthcare systems of the need for transforming an organizational culture to improve patient safety. The safety culture of an organization is the product of individual and group values, attitudes, perceptions, competencies, and patterns of behaviour that determine the commitment to the style and proficiency of an organization’s health and safety management [4]. Achieving a culture of safety requires an understanding of the values, beliefs and norms regarding health and safety within an organization, thus it is vital to unveil the underlying cultural factors present within the organization so that safety culture can be transformed [8, 9]. Frameworks, surveys and assessment tools have been designed over the past decade to measure and understand the type of culture that exists within healthcare organizations and to identify the areas of strengths and gaps [8, 10–14].

On a global basis, several international health bodies, such as the World Health Organization (WHO) and the Joint Commission International (JCI) advocate measuring safety culture within healthcare organizations as an effective strategy for sustainable safety improvement. A recent systematic review assessing the status of patient safety culture in the Arab countries called for the need to promote patient safety culture for improving patient safety in the Arab World [15].

There has been an intensive effort by the Ministry of Health (MoH) in Kuwait to ensure that the healthcare delivery meets the international standards. Accreditation Canada International (ACI) was commissioned in Kuwait to introduce a national accreditation program to be adopted by government hospitals and polyclinics [16]. On the other hand, the community pharmacy setting had less attention and therefore conducting this study is of utmost importance to explore the status of patient safety and to call for recommendations wherever necessary.

Few studies on safety culture were published in Kuwait [17–19]. However, none were surveyed among community pharmacists. To the best of our knowledge, this is the first study in the Middle East conducted in the community pharmacy setting to evaluate whether the practice of community pharmacists support patient safety and the extent to which safety is a strategic priority.

## Methods

### Aim of the study

The study aimed at evaluating whether the practice of community pharmacists in Kuwait supports patient safety and the extent to which safety is a strategic priority.

### Study design & settings

A descriptive cross-sectional study was conducted, using a pre-tested self-administered questionnaire. The questionnaire was distributed among pharmacists who work in community pharmacies from the five governorates of Kuwait.

### Sample size calculation and sampling strategy

Due to the unavailability of a list with the names and addresses of all community pharmacists, a list of the entire community pharmacies in the country was initially obtained from MoH. At the time of the study, a total of 454 community pharmacies were available and were scattered across the five health governorates in Kuwait [Capital (48), Hawalli (160), Jahra (48), Farwaniya (89), Ahmadi (109)]. The sample size was calculated based on the results of a previous similar study in Kuwait, where the response distribution of the main questions were 38% [18]. Minimum sample size of 202 was required at 95% confidence interval and 5% margin of error (Raosoft® software). Assuming a response rate of 80%, larger sample was targeted. The community pharmacies were first stratified at the level of the governorates. Then, a proportional percentage of the number of pharmacies to be selected from each governorate was calculated. The selection of pharmacies from the original list was carried out at the level of each governorate using systematic random sampling.

### Study tool

The Pharmacy Survey on Patient Safety Culture (PSOPSC), developed by the Agency for Healthcare Research and Quality (AHRQ); community pharmacy version, was used to collect data for the study [20] (Additional file 1). It includes 36 items that measure 11 dimensions of patient safety culture. In addition, the survey includes 3 items about the frequency of documenting mistakes, 3 items about respondent’s demographics, an overall rating question and a section for open-ended comments. Additional questions were added to the demographic section of the original questionnaire. The PSOPSC is a validated tool, and the reliability of its dimensions and their items have been proven [20]. The items for the parts were measured using the 5-point Likert response scale of agreement (Strongly Disagree to Strongly Agree) or frequency (Never to Always). The questionnaire was distributed in its original language (English).

### Ethics approval

Ethical clearance was sought from the Standing Committee for Coordination of Health and Medical Research, MoH and the Health Science Centre (HSC) Ethics Committee for Student Research, Kuwait University. Data was collected over a period of 5 months (January to May 2017).

### Inclusion and exclusion criteria

The study included pharmacists working in the community pharmacies, including the pharmacies of the cooperative societies. Other workers such as pharmacy helpers, cashiers and cleaners were excluded.

### Sample recruitment and data collection

Initially, a pilot study was carried out to ensure that the questions were clear and reflect the objectives of the study. Ten community pharmacies were selected randomly from the original list. Community pharmacists were invited verbally to participate. Before distributing the questionnaires, aim and objectives of the study were fully explained. If the pharmacy employed more than one pharmacist, then only one was invited to participate. A consent form was given to those who were willing to participate to assure their participation is voluntary. All collected information were kept confidential and anonymous. The questionnaire was collected back on the same day.

### Data analysis

The Statistical Package for Social Science (SPSS) software, version 24 was used for analysing the data. Data from closed-ended questions were coded and entered into the SPSS. The respondents’ demographics were presented using descriptive statistics; frequency/percentages, mean ± standard deviation (SD) and median or interquartile range (IQR). Calculation of composite frequencies for the 11 patient safety dimensions was carried out according to the instructions of the user’s guide published by the AHRQ [20].

The pharmacists’ perception about patient safety culture in their organizations is critical to achieve patient safety standards. Therefore, the study focused on measuring the positive response rate (PRR) as it illustrates pharmacist’s points of view on the different domains of patient safety culture in their setting. The PRR of the survey questions was calculated using the formula by the User’s Guide of PSOPSC [20]. Responses like “Does Not Apply/ Don’t Know” and missing responses were excluded when displaying the percentage of responses to the survey items. Chi square and Fisher’s exact testes were used, with a significance level of *p* < 0.05.

## Results

A total of 255 community pharmacists from the five governorates were approached to participate in the study, of whom 253 have returned the completed questionnaire, giving a response rate of 99%. The sociodemographic characteristics of the participants are shown in Table 1.

**Table 1:** Socio-demographic characteristics of participants (*n* = 253)

	n	(%)
Gender		
Male	182	(71.9)
Female	71	(28.1)
Age (years)		
20–29	37	(14.6)
30–39	122	(48.2)
40–49	77	(30.4)
≥ 50	17	(6.7)
Mean (SD)	37.4	(7.0)
Nationality		
Egyptian	175	(69.2)
Syrian	36	(14.2)
Jordanian	26	(10.3)
Indian	10	(4.0)
Pakistani	5	(2.0)
Palestinian	1	(0.4)
Years of experience		
< 5	36	(14.2)
6–10	52	(20.6)
11–15	71	(28.1)
16–20	61	(24.1)
> 20	33	(13.0)
Median (IQR)	14	(9–18)
Country of graduation		
Egypt	176	(69.6)
Syria	30	(11.9)
Jordan	31	(12.3)
India	10	(4.0)
Pakistan	5	(2.0)
Palestine	1	(0.4)
Last degree in Pharmacy		
Bachelor	234	(92.5)
Pharm D	7	(2.8)
Master	11	(4.3)
Other	1	(0.4)
Years of experience in the present Pharmacy		
< 6 months	11	(4.3)
6 months to < 1 year	26	(10.3)
1 year to < 3 years	89	(35.2)
3 years to < 6 years	65	(25.7)
6 years to > 12 years	46	(18.2)
≥ 12 years	16	(6.3)
Working hours per week in this pharmacy		
32–40	28	(11.1)
> 40	225	(88.9)
Governorate		
Capital	27	(10.7)
Hawalli	89	(35.2)
Farwaniya	50	(19.8)
Jahra	27	(10.7)
Ahmadi	60	(23.7)

*SD* standard deviation, *IQR* interquartile range

### Safety culture assessment across all the community pharmacies in Kuwait

The pharmacists’ perception about patient safety culture in their organizations is shown in Table 2.

**Table 2:** Positive response rate (PRR) of individual items and dimensions across all community pharmacies (*n* = 253)

	Responses	
	Total	PRR
	N	n (%)
1. Physical Space and Environment (dimension positivity 88.2%)		
A1. This pharmacy is well organized	251	233 (92.8)
A5. This pharmacy is free of clutter/untidiness	253	231 (91.3)
A7. The physical layout of this pharmacy supports good workflow	251	202 (80.5)
2. Team Work (dimension positivity 96.8%)		
A2. Staff treat each other with respect	253	247 (97.6)
A4. Staff in this pharmacy clearly understand their roles & responsibilities	252	241 (95.6)
A9. Staff work together as an effective team	253	246 (97.2)
3. Staff training and Skills (dimension positivity 89.5%)		
A3. Pharmacy assistants/helpers in this pharmacy receive the training they need to do their jobs	253	200 (79.1)
A6. Staff in this pharmacy have the skills they need to do their jobs well	253	242 (95.7)
A8. Staff who are new to this pharmacy receive adequate orientation	248	223 (89.9)
A10. Staff get enough training from this pharmacy	252	209 (82.9)
4. Communication Openness (dimension positivity 84.4%)		
B1. Staff ideas and suggestions are valued in this pharmacy	252	196 (77.8)
B5. Staff feel comfortable asking questions when they are unsure about something	253	223 (88.1)
B10. It is easy for staff to speak up to their pharmacy manager (chief pharmacist) or pharmacy owner about patient safety concerns in this pharmacy	251	219 (87.3)
5. Patient Counselling (dimension positivity 90.9%)		
B2. Pharmacists in this pharmacy encourage patients to talk about their medications	253	226 (89.3)
B7. Our pharmacists spend enough time talking to patients about how to use their medications	253	234 (92.5)
B11. Our pharmacists tell patients important information about their new prescriptions	253	230 (90.9)
6. Staffing, Work Pressure, and Pace (dimension positivity 49.7%)		
B3. Staff take adequate breaks during their shifts	253	107 (42.3)
B9. We feel rushed when processing prescriptions (**R**)	252	97 (38.5)
B12. We have enough staff to handle the workload	252	210 (83.3)
B16. Interruptions/distractions in this pharmacy (from phone calls, faxes, customers, etc.) make it difficult for staff to work accurately (**R**)	251	68 (27.1)
7. Communication about Prescriptions across Shifts (dimension positivity 74.6%)		
B4. We have clear expectations about exchanging important prescription information across shifts	251	185 (73.7)
B6. We have standard procedures for communicating prescription information across shifts	250	190 (76.0)
B14. The status of problematic prescriptions is well communicated across shifts	250	185 (74.0)
8. Communication about Mistakes (dimension positivity 81.8%)		
B8. Staff in this pharmacy discuss mistakes	253	212 (83.8)
B13. When patient safety issues occur in this pharmacy, staff discuss them	252	206 (81.7)
B15. In this pharmacy, we talk about ways to prevent mistakes from happening again	252	201 (79.8)
9. Response to Mistakes (dimension positivity 85.3%)		
C1. Staff are treated fairly when they make mistakes	251	235 (93.6)
C4. This pharmacy helps staff learn from their mistakes rather than punishing them	251	234 (93.2)
C7. We look at staff actions and the way we do things to understand why mistakes happen in this pharmacy	252	220 (87.3)
C8. Staff feel like their mistakes are held against them (**R**)	247	186 (75.3)
10. Organizational Learning - Continuous Improvement (dimension positivity 93.2%)		
C2. When a mistake happens, we try to figure out what problems in the work process led to the mistake	252	237 (94.0)
C5. When the same mistake keeps happening, we change the way we do things	249	225 (90.4)
C10. Mistakes have led to positive changes in this pharmacy	250	238 (95.2)
11. Overall Perceptions of Patient Safety (dimension positivity 87.5%)		
C3. This pharmacy places more emphasis on sales than on patient safety (**R**)	248	175 (70.6)
C6. This pharmacy is good at preventing mistakes	253	242 (95.7)
C9. The way we do things in this pharmacy reflects a strong focus on patient safety	251	241 (96.0)

(R): Negatively worded items were reversed coded

The standards of optimal patient safety were observed in the dimensions of “Teamwork” which scored the highest PRR (96.8%), followed by the “Organizational Learning-Continuous Improvement” and “Patient Counselling” domains which scored 93.2 and 90.9%, respectively. In general, there were more positive than negative responses to individual survey items and the positive responses ranged from 27.1 to 97.6%. An overall PRR of 83.8% was scored for all the 36 items. Table 2 displays the detailed scores.

Pharmacists’ overall perceptions of patient safety in their pharmacies were assessed by 3 items. The participants reported that the patient safety is never forgone to achieve more sales (*n* = 175; 70.6%); patient safety is a priority (*n* = 241; 96.0%); and pharmacy is good at preventing mistakes (*n* = 242; 95.7%). The pharmacists were also asked about the frequency of mistakes reporting within the pharmacy and tabulated as shown in Table 3**.** Regardless of the seriousness of the mistakes, they were never or rarely documented in most of the cases.

**Table 3:** Frequency of events reported by the community pharmacists (*n* = 253) from the five governorates

In this pharmacy, how often the following types of mistakes documented?	Never/Rarely n (%)	Sometimes n (%)	Most of the time/Always n (%)	PRR n (%)
D1. When a mistake reaches the patient and could cause harm but does not, how often is it documented?	143 (56.6)	60 (23.7)	44 (17.4)	44 (17.4)
D2. When a mistake reaches the patient but has no potential to harm the patient, how often is it documented?	147 (58.1)	60 (23.7)	39 (15.4)	39 (15.4)
D3. When a mistake that could have harmed the patient is corrected BEFORE the medication leaves the pharmacy, how often is it documented?	154 (60.8)	53 (20.9)	40 (15.8)	40 (15.8)

### Safety culture assessment at the level of governorates in Kuwait

There were statistically significant differences between the governorates in the dimensions relevant to pharmacy’s physical layout (A7), staff training (A3 and A10), patient counselling (B7), work pressure and pace (B3, B9 and B16), communication about mistakes (B13), and overall perception about the pharmacy (C3) **(**Table 4**)**. Regardless, a trend of similarity was observed with some items in Farwaniya, Ahmadi and Jahra governorates, compared to the Capital and Hawalli (Table 4).

**Table 4:** Positive response rate (PRR) on individual items and dimensions at the level of the governorates

	Capital	Hawalli	Farwaniya	Jahra	Ahmadi	*p*-value
	Positive	Positive	Positive	Positive	Positive	
	n (%)	n (%)	n (%)	n (%)	n (%)	
1. Physical Space and Environment						
A1. This pharmacy is well organized	24 (92.3)	84 (95.5)	45 (90.0)	26 (96.3)	54 (90.0)	0.611^a^
A5. This pharmacy is free of clutter/untidiness	24 (88.9)	81 (91.0)	44 (88.0)	26 (96.3)	56 (93.3)	0.755^a^
A7. The physical layout of this pharmacy supports good workflow	25 (96.2)	83 (94.3)	37 (74.0)	18 (66.7)	39 (65.0)	**< 0.001** ^**b**^
Dimension positivity (%)	92.5	93.6	84.0	86.4	82.8	**0.002** ^**b**^
2. Team work						
A2. Staff treat each other with respect	27 (100.0)	84 (94.4)	50 (100.0)	27 (100.0)	59 (98.3)	0.312^a^
A4. Staff in this pharmacy clearly understand their roles and responsibilities	27 (100.0)	82 (93.2)	49 (98.0)	25 (92.6)	58 (96.7)	0.487^a^
A9. Staff work together as an effective team	27 (100.0)	86 (96.6)	49 (98.0)	27 (100.0)	57 (95.0)	0.751^a^
Dimension positivity (%)	100	94.7	98.7	97.5	96.7	0.087^a^
3. Staff Training and Skills						
A3. Pharmacy assistants/helpers in this pharmacy receive the training they need to do their jobs	26 (96.3)	85 (95.5)	27 (54.0)	22 (81.5)	40 (66.7)	**< 0.001** ^**b**^
A6. Staff in this pharmacy have the skills they need to do their jobs well	25 (92.6)	87 (97.8)	48 (96.0)	27 (100.0)	55 (91.7)	0.275^a^
A8. Staff who are new to this pharmacy receive adequate orientation	24 (92.3)	82 (94.3)	45 (93.8)	23 (85.2)	49 (81.7)	0.100^a^
A10. Staff get enough training from this pharmacy	27 (100.0)	87 (97.8)	30 (61.2)	21 (77.8)	44 (73.3)	**< 0.001** ^**b**^
Dimension positivity (%)	95.3	96.4	76.3	86.1	78.4	**< 0.001** ^**b**^
4. Communication Openness						
B1. Staff ideas and suggestions are valued in this pharmacy	20 (74.1)	75 (85.2)	37 (74.0)	20 (74.1)	44 (73.3)	0.360^b^
B5. Staff feel comfortable asking questions when they are unsure about something	25 (92.6)	73 (82.0)	44 (88.0)	25 (92.6)	56 (93.3)	0.218^b^
B10. It is easy for staff to speak up to their pharmacy manager (chief pharmacist) or pharmacy owner about patient safety concerns in this pharmacy	24 (92.3)	73 (83.0)	45 (90.0)	23 (85.2)	54 (90.0)	0.573^b^
Dimension positivity (%)	86.3	83.4	84.0	84.0	85.5	0.960^b^
5. Patient Counselling						
B2. Pharmacists in this pharmacy encourage patients to talk about their medications	24 (88.9)	74 (83.1)	45 (90.0)	26 (96.3)	57 (95.0)	0.136^b^
B7. Our pharmacists spend enough time talking to patients about how to use their medications	25 (92.6)	76 (85.4)	48 (96.0)	27 (100.0)	58 (96.7)	**0.035** ^**a**^
B11. Our pharmacists tell patients important information about their new prescriptions	24 (88.9)	75 (84.3)	47 (94.0)	27 (100.0)	57 (95.0)	0.056^a^
Dimension positivity (%)	90.1	84.3	93.3	98.8	95.6	**< 0.001** ^**b**^
6. Staffing, Work Pressure, and Pace						
B3. Staff take adequate breaks during their shifts	16 (59.3)	73 (82.0)	13 (26.0)	1 (3.7)	4 (6.7)	**< 0.001** ^**b**^
B9. We feel rushed when processing prescriptions **(R)**	5 (18.5)	14 (15.7)	26 (52.0)	17 (63.0)	35 (59.3)	**< 0.001** ^**b**^
B12. We have enough staff to handle the workload	23 (85.2)	71 (80.7)	42 (84.0)	24 (88.9)	50 (83.3)	0.890^b^
B16. Interruptions/distractions in this pharmacy (from phone calls, faxes, customers, etc.) make it difficult for staff to work accurately **(R)**	3 (12.0)	2 (2.2)	21 (42.0)	13 (48.1)	29 (48.3)	**< 0.001** ^**b**^
Dimension positivity (%)	43.8	45.2	51.0	50.9	49.4	0.550^b^
7. Communication About Prescriptions Across Shifts						
B4. We have clear expectations about exchanging important prescription information across shifts	22 (81.5)	67 (76.1)	33 (67.3)	19 (70.4)	44 (73.3)	0.682^b^
B6. We have standard procedures for communicating prescription information across shifts	22 (81.5)	73 (83.0)	36 (75.0)	18 (66.7)	41 (68.3)	0.197^b^
B14. The status of problematic prescriptions is well communicated across shifts	20 (74.1)	67 (76.1)	39 (81.3)	17 (63.0)	42 (70.0)	0.444^b^
Dimension positivity (%)	79.0	78.4	74.5	66.7	70.5	0.131^b^
8. Communication About Mistakes						
B8. Staff in this pharmacy discuss mistakes	22 (81.5)	72 (80.9)	43 (86.0)	24 (88.9)	51 (85.0)	0.842^b^
B13. When patient safety issues occur in this pharmacy, staff discuss them	24 (88.9)	63 (70.8)	42 (84.0)	24 (88.9)	53 (89.8)	**0.019** ^**b**^
B15. In this pharmacy, we talk about ways to prevent mistakes from happening again	26 (96.3)	71 (79.8)	39 (79.6)	22 (81.5)	43 (71.7)	0.133^b^
Dimension positivity (%)	88.9	77.2	83.2	86.4	82.2	0.093^b^
9. Response to Mistakes						
C1. Staff are treated fairly when they make mistakes	23 (88.5)	83 (93.3)	47 (95.9)	24 (88.9)	58 (96.7)	0.405^a^
C4. This pharmacy helps staff learn from their mistakes rather than punishing them	25 (92.6)	82 (93.2)	46 (93.9)	26 (96.3)	55 (91.7)	0.973^a^
C7. We look at staff actions and the way we do things to understand why mistakes happen in this pharmacy	24 (88.9)	83 (93.3)	41 (83.7)	24 (88.9)	48 (80.0)	0.169^a^
C8. Staff feel like their mistakes are held against them **(R)**	21 (77.8)	63 (74.1)	34 (70.8)	24 (88.9)	44 (73.3)	0.482^b^
Dimension positivity (%)	87.0	85.3	86.1	90.8	85.4	0.607^b^
10. Organizational Learning - Continuous Improvement						
C2. When a mistake happens, we try to figure out what problems in the work process led to the mistake	24 (88.9)	85 (95.5)	46 (93.9)	26 (96.3)	56 (93.3)	0.766^a^
C5. When the same mistake keeps happening, we change the way we do things	24 (24.3)	79 (89.8)	40 (81.6)	25 (92.6)	58 (96.6)	0.132^a^
C10. Mistakes have led to positive changes in this pharmacy	27 (100.0)	82 (93.2)	46 (95.8)	26 (96.3)	57 (95.0)	0.807^a^
Dimension positivity (%)	71.1	92.8	90.4	95.1	95.0	0.522^b^
11. Overall Perceptions of Patient Safety						
C3. This pharmacy places more emphasis on sales than on patient safety **(R)**	22 (84.6)	70 (82.4)	31 (62.0)	17 (63.0)	35 (58.3)	**0.005** ^**b**^
C6. This pharmacy is good at preventing mistakes	25 (92.6)	85 (95.5)	49 (98.0)	26 (96.3)	57 (95.0)	0.858^a^
C9. The way we do things in this pharmacy reflects a strong focus on patient safety	27 (100.0)	83 (94.3)	49 (98.0)	25 (92.6)	58 (96.7)	0.603^a^
Dimension positivity (%)	92.4	90.7	86	84	83.3	0.070^b^

(R): Negatively worded items were reversed coded

*p*-values were generated using ^a^Fisher’s exact test and ^b^Pearson chi-square test

Significant numbers from the statistical tests were presenetd in bold

There was significantly (*p* < 0.001) less PRR observed in Farwaniya, Ahmadi and Jahra in terms of the adequacy of breaks during shifts compared to the Capital and Hawalli. More pharmacists in the Capital and Hawalli governorates felt (p < 0.001) rushed when processing prescriptions or were bothered with frequent interruptions. With respect to the patient counselling time, pharmacists in Hawalli governorates showed significantly (*p* = 0.035) less PRR than pharmacists in other governorates. Pharmacists in Hawalli also showed a significantly (*p* = 0.019) less PRR regarding the communication and discussion of any mistakes happened in the pharmacy (Table 4).

Regarding the perception of patient safety, although most of the pharmacists in every governorate disagreed that the pharmacies place more emphasis on sales rather than patient safety (C3), the disagreement was significantly (*p* = 0.005) less among pharmacists working in Farwaniya, Ahmadi and Jahra.

### Assessment of community pharmacies patient safety culture according to the pharmacists’ years of experience

The PRR for individual items, as well as the average rates for the 11 PSOPSC dimensions, were computed separately for the three groups of pharmacists according to their years of experience in the present community pharmacy (< 3 years, 3 to 5 years, and ≥ 6 years) (Table 5). Pharmacists having experience of 6 years or more significantly thought that both pharmacy assistants (A3) and staff members (A10) receive adequate training (*p* = 0.005 and *p* = 0.004, respectively) compared with those with less experience. Also, the average rate for “Staff Training and Skills” dimension was significantly higher among those with experience of 6 years or more compared to the less experienced groups (*p* = 0.001). Pharmacists with experience of 6 years or more believed that the staff members take adequate breaks during their shifts compared with those with less experience (B3, *p* < 0.001). The PRR for the items “we feel rushed when processing prescriptions (B9)” (p = 0.005) and “interruptions/distractions in this pharmacy make it difficult for staff to work accurately (B16)” (p = 0.001) was significantly lower among pharmacists with more than 6 years of experience compared to the less experienced groups.

**Table 5:** Positive response rate (PRR) for individual items/dimension according to pharmacists’ experience in their present workplace

	< 3 years	3- < 6 years	≥6 years	*p*-value
	Positive	Positive	Positive	
	n (%)	n (%)	n (%)	
1. Physical Space and Environment				
A1. This pharmacy is well organized	117 (92.9)	59 (93.7)	57 (91.9)	0.902^a^
A5. This pharmacy is free of clutter/untidiness	117 (92.9)	58 (89.2)	56 (90.3)	0.667^b^
A7. The physical layout of this pharmacy supports good workflow	97 (77.0)	53 (81.5)	52 (86.7)	0.288^b^
Total	331 (87.6)	170 (88.1)	165 (89.7)	0.766^b^
2. Teamwork				
A2. Staff treat each other with respect	123 (97.6)	64 (98.5)	60 (96.8)	0.760^a^
A4. Staff in this pharmacy clearly understand their roles and responsibilities	121 (96.0)	62 (96.9)	58 (95.2)	0.662^a^
A9. Staff work together as an effective team	122 (96.8)	65 (100.0)	59 (95.2)	0.245^a^
Total	366 (96.8)	191 (98.5)	186 (95.2)	0.187^b^
3. Staff Training and Skills				
A3. Pharmacy assistants/helpers in this pharmacy receive the training they need to do their jobs	93 (73.8)	49 (75.4)	58 (93.5)	**0.005** ^**b**^
A6. Staff in this pharmacy have the skills they need to do their jobs well	120 (95.2)	62 (95.4)	60 (96.8)	0.999^a^
A8. Staff who are new to this pharmacy receive adequate orientation	112 (90.3)	58 (90.6)	53 (88.3)	0.894^b^
A10. Staff get enough training from this pharmacy	98 (78.4)	51 (78.5)	60 (96.8)	**0.004** ^**b**^
Total	423 (84.4)	220 (84.9)	231 (93.9)	**0.001** ^**b**^
4. Communication Openness				
B1. Staff ideas and suggestions are valued in this pharmacy	94 (75.2)	53 (81.5)	49 (79.0)	0.586^b^
B5. Staff feel comfortable asking questions when they are unsure about something	113 (89.7)	55 (84.6)	55 (88.7)	0.583^b^
B10. It is easy for staff to speak up to their pharmacy manager (chief pharmacist) or pharmacy owner about patient safety concerns in this pharmacy	110 (87.3)	52 (81.3)	57 (93.4)	0.124^b^
Total	317 (84.1)	160 (82.5)	161 (87.0)	0.462^b^
5. Patient Counselling				
B2. Pharmacists in this pharmacy encourage patients to talk about their medications	117 (92.9)	57 (87.7)	52 (83.9)	0.152^b^
B7. Our pharmacists spend enough time talking to patients about how to use their medications	118 (93.7)	60 (92.3)	56 (90.3)	0.707^a^
B11. Our pharmacists tell patients important information about their new prescriptions	117 (92.9)	57 (87.7)	56 (90.3)	0.492^b^
Total	352 (93.1)	174 (89.2)	164 (88.2)	0.101^b^
6. Staffing, Work Pressure, and Pace				
B3. Staff take adequate breaks during their shifts	37 (29.4)	25 (38.5)	45 (72.6)	**< 0.001** ^**b**^
B9. We feel rushed when processing prescriptions **(R)**	59 (47.2)	24 (36.9)	14 (22.6)	**0.005** ^**b**^
B12. We have enough staff to handle the workload	107 (84.9)	53 (82.8)	50 (80.6)	0.754^b^
B16. Interruptions/distractions in this pharmacy (from phone calls, faxes, customers, etc.) make it difficult for staff to work accurately **(R)**	44 (35.2)	18 (28.1)	6 (9.7)	**0.001** ^**b**^
Total	247 (49.2)	120 (46.5)	115 (46.4)	0.680^b^
7. Communication About Prescriptions Across Shifts				
B4. We have clear expectations about exchanging important prescription information across shifts	93 (73.8)	43 (67.2)	49 (80.3)	0.249^b^
B6. We have standard procedures for communicating prescription information across shifts	94 (75.8)	46 (71.9)	50 (80.6)	0.513^b^
B14. The status of problematic prescriptions is well communicated across shifts	92 (74.2)	47 (73.4)	46 (74.2)	0.993^b^
Total	279 (74.6)	136 (70.8)	145 (78.4)	0.243^b^
8. Communication About Mistakes				
B8. Staff in this pharmacy discuss mistakes	104 (82.5)	53 (81.5)	55 (88.7)	0.474^b^
B13. When patient safety issues occur in this pharmacy, staff discuss them	106 (84.1)	52 (80.0)	48 (78.7)	0.608^b^
B15. In this pharmacy, we talk about ways to prevent mistakes from happening again	98 (77.8)	50 (78.1)	53 (85.5)	0.434^b^
Total	308 (81.5)	155 (79.9)	156 (84.3)	0.525^b^
9. Response to Mistakes				
C1. Staff are treated fairly when they make mistakes	118 (94.4)	59 (92.2)	58 (93.5)	0.843^a^
C4. This pharmacy helps staff learn from their mistakes rather than punishing them	118 (93.7)	59 (93.7)	57 (91.9)	0.897^a^
C7. We look at staff actions and the way we do things to understand why mistakes happen in this pharmacy	110 (87.3)	55 (85.9)	55 (88.7)	0.897^b^
C8. Staff feel like their mistakes are held against them **(R)**	95 (76.6)	51 (79.7)	40 (67.8)	0.278^b^
Total	441 (88.0)	224 (87.8)	210 (85.7)	0.652^b^
10. Organizational Learning - Continuous Improvement				
C2. When a mistake happens, we try to figure out what problems in the work process led to the mistake	118 (94.4)	60 (92.3)	59 (95.2)	0.782^a^
C5. When the same mistake keeps happening, we change the way we do things	114 (91.2)	57 (89.1)	54 (90.0)	0.890^b^
C10. Mistakes have led to positive changes in this pharmacy	120 (96.8)	58 (90.6)	60 (96.8)	0.188^a^
Total	352 (94.1)	175 (90.7)	173 (94.0)	0.267^b^
11. Overall Perceptions of Patient Safety				
C3. This pharmacy places more emphasis on sales than on patient safety **(R)**	84 (67.7)	48 (73.8)	43 (72.9)	0.617^b^
C6. This pharmacy is good at preventing mistakes	121 (96.0)	63 (96.9)	58 (93.5)	0.663^a^
C9. The way we do things in this pharmacy reflects a strong focus on patient safety	119 (95.2)	62 (95.4)	60 (98.4)	0.696^a^
Total	324 (86.4)	173 (88.7)	161 (88.5)	0.659^b^

(R): Negatively worded items were reversed coded

*p*-values were generated using ^a^Fisher’s exact test and ^b^Pearson chi-square test

Significant numbers from the statistical tests were presenetd in bold

### Assessment of community pharmacies patient safety culture according to the number of working hours per week of the pharmacists in the present community pharmacy

Pharmacists who worked 40 h per week or less scored significantly (*p* = 0.024) higher average rate for the “Staff Training and Skills” dimension compared to those who worked more than 40 h per week (Table 6). On the other hand, the average PRR for the dimensions “Patient Counselling” and “Response to Mistakes” were significantly lower among those who worked 40 h per week or less compared to their counterparts (*p* = 0.010 and *p* = 0.013, respectively). The PRR for the items “we feel rushed when processing prescriptions (B9)” and “interruptions/distractions in this pharmacy make it difficult for staff to work accurately (B16)” in the dimension “Staffing, Work Pressure, and Pace” were significantly lower among those pharmacists who worked less than 40 h per week compared to those who worked for longer hours, *p* = 0.048).

**Table 6:** Positive response rate (PRR) for individual items/dimensions according to pharmacists’ working hours per week

	32–40 h per week	> 40 h per week	*p*-value
	Positive	Positive	
	n (%)	n (%)	
1. Physical Space and Environment			
A1. This pharmacy is well organized	25 (92.6)	208 (92.9)	0.999^a^
A5. This pharmacy is free of clutter/untidiness	26 (92.9)	205 (91.1)	0.999^a^
A7. The physical layout of this pharmacy supports good workflow	24 (88.9)	178 (79.5)	0.243^b^
Total	75 (91.5)	591 (87.8)	0.333^b^
2. Teamwork			
A2. Staff treat each other with respect	28 (100.0)	219 (97.3)	0.999^a^
A4. Staff in this pharmacy clearly understand their roles and responsibilities	26 (96.3)	215 (95.6)	0.999^a^
A9. Staff work together as an effective team	28 (100.0)	218 (96.9)	0.999^a^
Total	82 (98.8)	652 (96.6)	0.503^a^
3. Staff Training and Skills			
A3. Pharmacy assistants/helpers in this pharmacy receive the training they need to do their jobs	26 (92.9)	174 (77.3)	0.057^b^
A6. Staff in this pharmacy have the skills they need to do their jobs well	27 (96.4)	215 (95.6)	0.999^a^
A8. Staff who are new to this pharmacy receive adequate orientation	25 (92.6)	198 (89.6)	0.999^a^
A10. Staff get enough training from this pharmacy	26 (92.9)	183 (81.7)	0.185^a^
Total	104 (93.7)	770 (86.0)	**0.024** ^**b**^
4. Communication Openness			
B1. Staff ideas and suggestions are valued in this pharmacy	22 (81.5)	174 (77.3)	0.624^b^
B5. Staff feel comfortable asking questions when they are unsure about something	24 (85.7)	199 (88.4)	0.755^a^
B10. It is easy for staff to speak up to their pharmacy manager (chief pharmacist) or pharmacy owner about patient safety concerns in this pharmacy	23 (82.1)	196 (87.9)	0.373^a^
Total	69 (83.1)	569 (84.5)	0.738^b^
5. Patient Counselling			
B2. Pharmacists in this pharmacy encourage patients to talk about their medications	23 (82.1)	203 (90.2)	0.196^a^
B7. Our pharmacists spend enough time talking to patients about how to use their medications	24 (85.7)	210 (93.3)	0.242^a^
B11. Our pharmacists tell patients important information about their new prescriptions	23 (82.1)	207 (92.0)	0.152^a^
Total	70 (83.3)	620 (91.9)	**0.010** ^**b**^
6. Staffing, Work Pressure, and Pace			
B3. Staff take adequate breaks during their shifts	19 (67.9)	88 (39.1)	**0.004** ^**b**^
B9. We feel rushed when processing prescriptions **(R)**	5 (17.9)	92 (41.1)	**0.017** ^**b**^
B12. We have enough staff to handle the workload	23 (82.1)	187 (83.5)	0.792^a^
B16. Interruptions/distractions in this pharmacy (from phone calls, faxes, customers, etc.) make it difficult for staff to work accurately **(R)**	3 (11.1)	65 (29.0)	**0.048** ^**b**^
Total	50 (45.0)	432 (48.2)	0.061^b^
7. Communication About Prescriptions Across Shifts			
B4. We have clear expectations about exchanging important prescription information across shifts	24 (85.7)	161 (72.2)	0.126^b^
B6. We have standard procedures for communicating prescription information across shifts	21 (77.8)	169 (75.8)	0.819^b^
B14. The status of problematic prescriptions is well communicated across shifts	20 (74.1)	165 (74.0)	0.993^b^
Total	65 (79.3)	495 (74.0)	0.300^b^
8. Communication About Mistakes			
B8. Staff in this pharmacy discuss mistakes	21 (75.0)	191 (84.9)	0.181^a^
B13. When patient safety issues occur in this pharmacy, staff discuss them	23 (82.1)	183 (81.7)	0.954^b^
B15. In this pharmacy, we talk about ways to prevent mistakes from happening again	24 (85.7)	177 (79.0)	0.406^b^
Total	68 (81.0)	551 (81.9)	0.837^b^
9. Response to Mistakes			
C1. Staff are treated fairly when they make mistakes	24 (88.9)	211 (94.2)	0.392^a^
C4. This pharmacy helps staff learn from their mistakes rather than punishing them	24 (85.7)	210 (94.2)	0.106^a^
C7. We look at staff actions and the way we do things to understand why mistakes happen in this pharmacy	25 (89.3)	195 (87.1)	0.999^a^
C8. Staff feel like their mistakes are held against them **(R)**	15 (55.6)	171 (77.7)	**0.012** ^**b**^
Total	88 (80.0)	787 (88.3)	**0.013** ^**b**^
10. Organizational Learning - Continuous Improvement			
C2. When a mistake happens, we try to figure out what problems in the work process led to the mistake	25 (89.3)	212 (94.6)	0.224^a^
C5. When the same mistake keeps happening, we change the way we do things	23 (85.2)	202 (91.0)	0.308^a^
C10. Mistakes have led to positive changes in this pharmacy	27 (96.4)	211 (95.0)	0.999^a^
Total	75 (90.4)	625 (93.6)	0.274^b^
11. Overall Perceptions of Patient Safety			
C3. This pharmacy places more emphasis on sales than on patient safety **(R)**	20 (74.1)	155 (70.1)	0.672^b^
C6. This pharmacy is good at preventing mistakes	25 (89.3)	217 (96.4)	0.109^a^
C9. The way we do things in this pharmacy reflects a strong focus on patient safety	26 (96.3)	215 (96.0)	0.999^a^
Total	71 (86.6)	587 (87.6)	0.791^b^

(R): Negatively worded items were reversed coded

*p*-values were generated using ^a^Fisher’s exact test and ^b^Pearson chi-square test

Significant numbers from the statistical tests were presenetd in bold

The overall grade of patient safety rating given by the pharmacists to their pharmacy was very high (Good 25.3%, Very good 50.2% and Excellent 20.6%). There were no significant differences in ratings according to the years of experience or the hours they work in the pharmacy.

## Discussion

This study is the first of its kind in Kuwait to report data on the community pharmacists’ perspectives of patient safety culture in their practice. Furthermore, the third publication of its kind using the PSOPSC to explore patient safety culture in the setting of community pharmacies [21, 22], following the piloting of the questionnaire in 60 US pharmacies approximately 6 years ago during the development of the study tool [23]. Although many questionnaires were developed to assess patient safety culture, they are generally focused toward healthcare organizations as a whole and are not customized to specific departments within an organization [13]. PSOPSC is a very comprehensive survey and community pharmacy specific.

The response rate in the present study was 99.0%, which was higher than that of previous studies [17–19, 24, 25], including the study in Malaysian retail pharmacies (93.52%) [21] and the AHRQ study (75.0%). The higher response rate in the study can be due to the fact that the questionnaires were distributed in-person by the researcher. This contrasts to online surveys used for data collection in the previous studies [25, 26]. Many community pharmacists were requested to complete the questionnaire during the overnight shifts when pharmacists did not have much customers, hence, they had sufficient time to fill out the questionnaire.

For the 36 items of the questionnaire, an overall mean of 83.3% was scored for patient safety culture which is higher than that in earlier studies conducted in Malaysia [21] and China [24] . This reflects that the pharmacists working at the community pharmacy understood the importance and values of patient safety in their practice. Findings in the current study also showed that there was a significant variability in the PRR values across the dimensions of patient safety. In line with other local [17–19] and international studies [21, 27], the highest PRR was seen in the “Teamwork” domain (96.8%), suggesting that the concept of teamwork is universally positive in the practice of pharmacy. This was followed by the domains of “Organizational Learning-Continuous Improvement” and “Patient Counselling” scoring 93.2 and 90.9% respectively, which highlight the existence of a learning culture among practicing pharmacists to improve the services which in turn can enhance the patient safety. In addition, patient counselling is the core of pharmacy practice in the community pharmacies in Kuwait and community pharmacists expressed their engagement and willingness to spend enough time with patients to discuss about their medications. With this respect, studies have shown that community pharmacists’ interventions can reflect positively on patients’ adherence to medications and subsequently their therapeutic outcomes [28].

The lowest PRR was given to the “Staffing, Work Pressure, and Pace” domain which scored 49.7%, close to the study from Malaysia [21]. In particular, the frequent interruptions or distractions in the pharmacy, feeling rushed when processing prescriptions and inadequate breaks during shifts were given the lowest PRR of 27.1, 38.5, and 42.3%, respectively. In addition, pharmacists working for more than 40 h per week had significantly lower PRR on the adequacy of breaks during shifts (Table 6). The environment of the pharmacy was a significant factor related to medication errors [29–31] and increased dispensing errors were reported where there was unfavourable working environment [32]. Taking this into account, all community pharmacists in Kuwait and elsewhere should be supported by a favourable workplace to allow them to work more efficiently.

At the level of governorates, there was a significant discrepancy in responses of community pharmacists from the five governorates on some items of the “Staffing, Work Pressure, and Pace” domains. Although the majority agreed that the pharmacy had enough staff, the Capital and Hawalli governorates scored the lowest in the items of pharmacists feeling rushed when processing prescriptions and having frequent interruptions, while Farwaniya, Jahra and Ahmadi had the lowest scores with regards to inadequate breaks during shifts. This could be partly explained by uneven proportion of the community pharmacy distribution across governorates as opposed to population number. For example, according to the last published Annual Health Report of Kuwait [33], Farwaniya was the most populous governorate, with a population size of 1,032,082, followed by Ahmadi, Hawalli, Capital and lastly Jahra. In comparison, the total number of community pharmacies in Farwaniya region was 89 (as compared to 160 in Hawalli, 109 in Ahmadi, 48 in Capital and 48 in Jahra) according to the data obtained during the preliminary fieldwork from the MoH, Kuwait in 2017. Therefore, results from the study suggest the need for accurate planning for the distribution of community pharmacies in Kuwait, and in other countries, to target the population size in each region.

The “Communication about Prescriptions across Shifts” and “Communication about Mistakes” dimensions showed relatively lower scores of 74.6 and 81.8%, respectively, which indicates the need for improvement. In dispensing medications, effective communication is crucial to minimize drug related problems. Pharmacy staff should learn from each other’s by knowing their own responsibilities as well as engaging in discussing mistakes and providing feedback. The mutual understanding among working staff on patient safety issues and knowledge on sources of mistakes and ways of detecting and preventing them will enhance the patient safety [34]. Also, results from the study showed that regardless of their seriousness, there was a minimal documentation of mistakes carried out in the community pharmacies. The MoH in Kuwait should have a proactive role in setting specific policies and guidelines for strategies that should be followed when identifying and communicating mistakes, similar to other international communities [35].

The work experience level and length of working hours are closely related to the relative occurrence of the patient-related risk events [36–38]. This study tested pharmacists’ PRR on safety culture items in respect to experience years and working hours per week. Pharmacists with more work experience (a minimum of 6 years of experience) scored better PRR for items relevant to adequacy of training and breaks during shifts. This could be related to the fact that their seniority in work allowed them to master pharmacy workload and tasks more than those with less years of experience. In line with this, senior pharmacists in the study by Jia et al. [24] gave significantly higher PRR scores on the item of staff taking adequate breaks during shifts. These results suggest the importance of providing less experienced community pharmacists with adequate training and breaks during shifts to handle workload and to minimize errors.

### Study strengths and limitations

This study is the first in the community pharmacy setting in Kuwait and included a large heterogeneous sample of community pharmacists from all over the country. Thus, taking the overall response rate into account (99.0%), the study results can be considered as the representative of the target population of community pharmacists. The selection of the pharmacies was done randomly from the original list provided.

Taking into consideration that all of the community pharmacists in the study were non-citizens, authors think that the results of the study might have inflated the real picture of safety culture in community pharmacies in Kuwait. The use of questionnaires as data collection tool has a disadvantage of response bias [39] and authors think that the risk is higher in the study due to the fear of the impact of negative responses on the pharmacies’ reputation and consequently pharmacists’ job security. This could have been reduced if the survey method for data collection was combined with another method, such as observation (i.e. triangulation method) which would have been validated the data presented in the study [39]. Therefore, repeating the study with a triangulation approach would be a validation approach in future research. In addition, testing the impact of having low-rated domains of safety culture on patient safety was out of the scope of the current study. Hence, evaluating the consequences of weakness in certain safety culture domains on patient safety and the quality of healthcare services should be considered in future research.

The study has only included the responses of community pharmacists, hence the responses of pharmacists working in other settings, such as private or government hospitals where workflow and responsibilities might differ. Hence, results from this study cannot be generalized to pharmacists working in other settings.

## Conclusion

Understanding patient safety culture from the perspectives of community pharmacists can help identify areas of weakness and can support decision for improvements. Our results suggest that pharmacies in Kuwait should develop strategies and plans to improve aspects of patient safety such as communication about prescriptions throughout shifts and responses against mistakes and create a positive culture that encourages patient’s safety in this setting. Urgent attention should be given to the areas of weakness in the patient safety, which has been observed mainly in the dimension of Staffing, Work Pressure, and Pace. With this respect, stakeholders should undertake the required steps towards supporting community pharmacists with adequate number of staff and with organized less distractible environment in order to perform their work accurately without jeopardizing patient safety.

## Additional file

Additional File 1: Safety Culture Questionnaire, community pharmacy version. The PSOPSC, developed by the AHRQ is a self-administered validated tool that measures 11 dimensions (36 items) of patient safety culture. (DOC 210 kb)

## Abbreviations

ACI: Accreditation Canada International
ADE: adverse drug events
AHRQ: Agency for Healthcare Research and Quality
HSC: Health Science Centre
IOM: Institute of Medicine
IQR: Interquartile range
JCI: Joint Commission International
MoH: Ministry of Health
PRR: Positive response rate
PSOPSC: Pharmacy Survey on Patient Safety Culture
SD: standard deviation
SPSS: Statistical Package for Social Science
WHO: World Health Organization
