# Supplementary material for: Assessment of patient safety culture: a nationwide survey of community pharmacists in Kuwait
Source: BMC Health Serv Res. 2018 Nov 22;18:884. doi: 10.1186/s12913-018-3662-0 (PMC6251142; doi:10.1186/s12913-018-3662-0)
Supplement: Supplementary file 1 — Safety Culture Questionnaire, community pharmacy version. The PSOPSC, developed by the AHRQ is a self-administered validated tool that measures 11 dimensions (36 items) of patient safety culture. (DOC 210 kb) [file 12913_2018_3662_MOESM1_ESM.doc]

Community Pharmacy Survey on Patient Safety

This survey asks for your opinions about patient safety in this community pharmacy. Answer only about the pharmacy you are currently working in.

**► Patient safety** is the prevention of patient harm resulting from the processes of health care delivery. In the pharmacy setting, it means that:

- The right patient receives the right medication in the right dose at the right time by the right route, and
- The patient or caregiver understands the purpose and proper use of the medication.

**► *If a question does not apply to you or you don’t know the answer, please answer* “Does Not Apply or Don’t Know.”**

| SECTION A: Working in This Pharmacy | | | | | | |
| --- | --- | --- | --- | --- | --- | --- |
|  | | | | | | |
| ***Staff*** *means* ***EVERYONE who works in this community pharmacy,*** *including pharmacists, pharmacy assistants or helpers, etc*. | | | | | | |
| **How much do you agree or disagree with the following statements?** | **Strongly Disagree** ⯆ | **Disagree** ⯆ | **Neither**  **Agree nor Disagree ⯆** | **Agree** ⯆ | **Strongly Agree** ⯆ | **Does Not Apply or Don’t Know**  ⯆ |
| 1. This pharmacy is well organized | 1 | 2 | 3 | 4 | 5 | 9 |
| 1. Staff treat each other with respect | 1 | 2 | 3 | 4 | 5 | 9 |
| 1. Pharmacy assistants/helpers in this pharmacy receive the training they need to do their jobs | 1 | 2 | 3 | 4 | 5 | 9 |
| 1. Staff in this pharmacy clearly understand their roles and responsibilities | 1 | 2 | 3 | 4 | 5 | 9 |
| 1. This pharmacy is free of clutter/untidiness (فوضى) | 1 | 2 | 3 | 4 | 5 | 9 |
| 1. Staff in this pharmacy have the skills they need to do their jobs well | 1 | 2 | 3 | 4 | 5 | 9 |
| 1. The physical layout of this pharmacy supports good workflow | 1 | 2 | 3 | 4 | 5 | 9 |
| 1. Staff who are new to this pharmacy receive adequate orientation | 1 | 2 | 3 | 4 | 5 | 9 |
| 1. Staff work together as an effective team | 1 | 2 | 3 | 4 | 5 | 9 |
| 1. Staff get enough training from this pharmacy | 1 | 2 | 3 | 4 | 5 | 9 |

| SECTION B: Communication and Work Pace | | | | | | | | |
| --- | --- | --- | --- | --- | --- | --- | --- | --- |
|  | **How often do the following statements apply to this pharmacy?** | **Never** ⯆ | **Rarely** ⯆ | **Some-**  **times ⯆** | **Most of the time** ⯆ | **Always** ⯆ | **Does Not Apply or Don’t Know**  ⯆ |  |
|  | 1. Staff ideas and suggestions are valued in this pharmacy | 1 | 2 | 3 | 4 | 5 | 9 |  |
|  | 1. Pharmacists in this pharmacy encourage patients to talk about their medications | 1 | 2 | 3 | 4 | 5 | 9 |  |
|  | 1. Staff take adequate breaks during their shifts | 1 | 2 | 3 | 4 | 5 | 9 |  |
|  | 1. We have clear expectations about exchanging important prescription information across shifts | 1 | 2 | 3 | 4 | 5 | 9 |  |
|  | 1. Staff feel comfortable asking questions when they are unsure about something | 1 | 2 | 3 | 4 | 5 | 9 |  |
|  | 1. We have standard procedures for communicating prescription information across shifts | 1 | 2 | 3 | 4 | 5 | 9 |  |
|  | 1. Our pharmacists spend enough time talking to patients about how to use their medications | 1 | 2 | 3 | 4 | 5 | 9 |  |
|  | 1. Staff in this pharmacy discuss mistakes | 1 | 2 | 3 | 4 | 5 | 9 |  |
|  | 1. We feel rushed when processing prescriptions | 1 | 2 | 3 | 4 | 5 | 9 |  |
|  | 1. It is easy for staff to speak up to their pharmacy manager (chief pharmacist) or pharmacy owner about patient safety concerns in this pharmacy | 1 | 2 | 3 | 4 | 5 | 9 |  |
|  | 1. Our pharmacists tell patients important information about their new prescriptions | 1 | 2 | 3 | 4 | 5 | 9 |  |
|  | 1. We have enough staff to handle the workload | 1 | 2 | 3 | 4 | 5 | 9 |  |
|  | 1. When patient safety issues occur in this pharmacy, staff discuss them | 1 | 2 | 3 | 4 | 5 | 9 |  |
|  | 1. The status of problematic prescriptions is well communicated across shifts | 1 | 2 | 3 | 4 | 5 | 9 |  |
|  | 1. In this pharmacy, we talk about ways to prevent mistakes from happening again | 1 | 2 | 3 | 4 | 5 | 9 |  |
|  | 1. Interruptions/distractions in this pharmacy (from phone calls, faxes, customers, etc.) make it difficult for staff to work accurately | 1 | 2 | 3 | 4 | 5 | 9 |  |

| SECTION C: Patient Safety and Response to Mistakes |
| --- |

***A mistake is any type of medication error, mistake, incident, or quality-related event, regardless of whether or not it reaches the patient or results in patient harm.*** *It may be related to, or include: prescribing, dispensing, counselling, monitoring (use of medication), unsafe conditions or procedures in the pharmacy, etc.*

| **How much do you agree or disagree with the following statements?** | **Strongly Disagree** ⯆ | **Disagree** ⯆ | **Neither**  **Agree nor Disagree ⯆** | **Agree** ⯆ | **Strongly Agree** ⯆ | **Does Not Apply or Don’t Know**  ⯆ |
| --- | --- | --- | --- | --- | --- | --- |
| 1. Staff are treated fairly when they make mistakes | 1 | 2 | 3 | 4 | 5 | 9 |
| 1. When a mistake happens, we try to figure out what problems in the work process led to the mistake | 1 | 2 | 3 | 4 | 5 | 9 |
| 1. This pharmacy places more emphasis on sales than on patient safety | 1 | 2 | 3 | 4 | 5 | 9 |
| 1. This pharmacy helps staff learn from their mistakes rather than punishing them | 1 | 2 | 3 | 4 | 5 | 9 |
| 1. When the same mistake keeps happening, we change the way we do things | 1 | 2 | 3 | 4 | 5 | 9 |
| 1. This pharmacy is good at preventing mistakes | 1 | 2 | 3 | 4 | 5 | 9 |
| 1. We look at staff actions **and** the way we do things to understand why mistakes happen in this pharmacy | 1 | 2 | 3 | 4 | 5 | 9 |
| 1. Staff feel like their mistakes are held against them | 1 | 2 | 3 | 4 | 5 | 9 |
| 1. The way we do things in this pharmacy reflects a strong focus on patient safety | 1 | 2 | 3 | 4 | 5 | 9 |
| 1. Mistakes have led to positive changes in this pharmacy | 1 | 2 | 3 | 4 | 5 | 9 |

| SECTION D: Documenting Mistakes |
| --- |

**In this pharmacy, how often are the following types of mistakes documented (in writing OR tracked electronically)**?

|  | **Never documented** ⯆ | | **Rarely documented** ⯆ | | **Sometimes documented ⯆** | | **Most of the time documented** ⯆ | | **Always documented** ⯆ | | **Does Not Apply or Don’t Know**  ⯆ |  |
| --- | --- | --- | --- | --- | --- | --- | --- | --- | --- | --- | --- | --- |
| 1. When a mistake reaches the patient and **could cause harm but does not**, how often is it documented? | | 1 | | 2 | | 3 | | 4 | | 5 | 9 | |
| 2. When a mistake reaches the patient but has **no potential to harm** the patient, how often is it documented? | | 1 | | 2 | | 3 | | 4 | | 5 | 9 | |
| 3. When a mistake **that could have harmed the patient is corrected BEFORE the medication leaves the pharmacy,** how often is it documented? | | 1 | | 2 | | 3 | | 4 | | 5 | 9 | |

| SECTION E: Overall Rating |
| --- |

1. **Think back on the survey topics and the definition of patient safety—dispensing the right medication accurately and making sure patients understand their medications and how to use them:**

**How do you rate this pharmacy on patient safety?**

| **Poor**  **▼** | **Fair**  **▼** | **Good**  **▼** | **Very good**  **▼** | **Excellent**  **▼** |
| --- | --- | --- | --- | --- |
| 1 | 2 | 3 | 4 | 5 |

| SECTION F: Background Questions |
| --- |

1. **Age ………………..Years**

| 1. **Gender** 1 Male 2 Female 2. **Nationality** 1 Kuwaiti 2 Non-Kuwaiti (please specify)…………….. |
| --- |

1. **What is your LAST degree in pharmacy?**

1 Bachelor 2 Pharm D 3 Master 4 PhD 5 Other ………………………

1. **Country of graduation: ……………………………………………**
2. **Practice years ……………………………………………(years)**
3. **How long have you worked in this pharmacy**?

| 1 Less than 6 months |
| --- |
| 2 6 months to less than 1 year |
| 3 1 year to less than 3 years |
| 4 3 years to less than 6 years |
| 5 6 years to less than 12 years |
| 6 12 years or more |

1. Typically, how many hours per week do you work in this pharmacy?

|  | 1 1 to 16 hours per week | |
| --- | --- | --- |
|  | 2 17 to 31 hours per week | |
| 3 32 to 40 hours per week | |  |
| 4 More than 40 hours per week | |  |

1. **In which governorate is this pharmacy located?**

1 Capital 2 Hawalli 3 Farwaniya 4 Jahra 5 Ahmadi

| SECTION G: Your Comments |
| --- |

**Please feel free to write any comments about how things are done or could be done in your pharmacy that might affect patient safety.**

|  |
| --- |

***THANK YOU FOR COMPLETING THIS SURVEY.***
